# The Effects of Mirror Feedback during Target Directed Movements on Ipsilateral Corticospinal Excitability

**DOI:** 10.3389/fnhum.2017.00242

**Published:** 2017-05-11

**Authors:** Mathew Yarossi, Thushini Manuweera, Sergei V. Adamovich, Eugene Tunik

**Affiliations:** ^1^Graduate School of Biomedical Sciences, Rutgers Biomedical and Health SciencesNewark, NJ, USA; ^2^Department of Biomedical Engineering, New Jersey Institute of TechnologyNewark, NJ, USA; ^3^Department of Rehabilitation and Movement Sciences, Rutgers Biomedical Health SciencesNewark, NJ, USA; ^4^Department of Physical Therapy, Movement, and Rehabilitation Sciences, Northeastern UniversityBoston, MA, USA; ^5^Department of Bioengineering, Northeastern UniversityBoston, MA, USA; ^6^Department of Biology, Northeastern UniversityBoston, MA, USA; ^7^Department of Electrical and Computer Engineering, Northeastern UniversityBoston, MA, USA

**Keywords:** TMS, mirror feedback, target, action observation, virtual reality

## Abstract

Mirror visual feedback (MVF) training is a promising technique to promote activation in the lesioned hemisphere following stroke, and aid recovery. However, current outcomes of MVF training are mixed, in part, due to variability in the task undertaken during MVF. The present study investigated the hypothesis that movements directed toward visual targets may enhance MVF modulation of motor cortex (M1) excitability ipsilateral to the trained hand compared to movements without visual targets. Ten healthy subjects participated in a 2 × 2 factorial design in which feedback (veridical, mirror) and presence of a visual target (target present, target absent) for a right index-finger flexion task were systematically manipulated in a virtual environment. To measure M1 excitability, transcranial magnetic stimulation (TMS) was applied to the hemisphere ipsilateral to the trained hand to elicit motor evoked potentials (MEPs) in the untrained first dorsal interosseous (FDI) and abductor digiti minimi (ADM) muscles at rest prior to and following each of four 2-min blocks of 30 movements (B1–B4). Targeted movement kinematics without visual feedback was measured before and after training to assess learning and transfer. FDI MEPs were decreased in B1 and B2 when movements were made with veridical feedback and visual targets were absent. FDI MEPs were decreased in B2 and B3 when movements were made with mirror feedback and visual targets were absent. FDI MEPs were increased in B3 when movements were made with mirror feedback and visual targets were present. Significant MEP changes were not present for the uninvolved ADM, suggesting a task-specific effect. Analysis of kinematics revealed learning occurred in visual target-directed conditions, but transfer was not sensitive to mirror feedback. Results are discussed with respect to current theoretical mechanisms underlying MVF-induced changes in ipsilateral excitability.

## Introduction

The use of mirror visual feedback (MVF) for neurorehabilitation has proliferated in the 20 years since the landmark study by Ramachandran illustrated its application for phantom pain in amputee patients (Ramachandran and Rogers-Ramachandran, 1996). The reflection of movement of the unimpaired hand projected over the impaired hand is an attractive intervention for persons with severe hemiplegia due to stroke who have limited capacity to participate in traditional repetitive task-based therapy (Ramachandran and Altschuler, 2009). The application of MVF to improve motor deficits associated with hemiplegia due to stroke shows efficacy (Altschuler et al., 1999; Sathian et al., 2000; Sütbeyaz et al., 2007; Dohle et al., 2009; Thieme et al., 2012, 2013) and is now a recommended physical therapy treatment (Pollock et al., 2014).

Perhaps most important to the rehabilitation potential of MVF for hemiplegia is empirical data showing that MVF can facilitate activity of the sensorimotor cortex ipsilateral to the moving hand (for review, see Deconinck et al., 2015). Numerous investigations using transcranial magnetic stimulation (TMS) to probe corticospinal excitability (CSE) have indicated increased amplitude of motor evoked potentials (MEPs) in the muscles of the resting hand homologous to those in the active hand that were involved in the task (Garry et al., 2005; Funase et al., 2007; Nojima et al., 2012; Kumru et al., 2016). Ipsilateral sensorimotor cortex changes associated with MVF have also been measured as a change in signal laterality using EEG and MEG (Touzalin-Chretien and Dufour, 2008; Praamstra et al., 2009, 2011; Tominaga et al., 2009; Touzalin-Chretien et al., 2010; Debnath and Franz, 2016; Franz et al., 2016), and with fMRI, quantified as an increased BOLD response in sensorimotor areas ipsilateral to the moving hand (Michielsen et al., 2011a,b; Hamzei et al., 2012; Saleh et al., 2014).

It is worth noting, however, that a number of studies have failed to show a significant increase in ipsilateral M1 excitability evoked by MVF (Läppchen et al., 2012; Mehnert et al., 2013; Avanzino et al., 2014; Fritzsch et al., 2014; Ruddy et al., 2016). Moreover, recent reviews of clinical (Veerbeek et al., 2014) and neurophysiological (Deconinck et al., 2015) investigations revealed wide variation in effect sizes between studies. Differences in experimental designs, including the use of unimanual or bimanual movements, timing of assessment, type of visual feedback in control conditions, and movement task, make comparisons between MVF studies difficult. Careful examination of movement tasks used in previous investigations, limiting search parameters to those studies using TMS to directly assess ipsilateral motor cortex (M1) excitability changes resulting from MVF, revealed that for many tasks, visual feedback may not have been a strong prerequisite for accurate task completion. Such tasks include finger tapping/opposition (Garry et al., 2005; Avanzino et al., 2014), oscillatory movements (Funase et al., 2007; Senna et al., 2015), non-targeted ballistic movements (Reissig et al., 2015a; Ruddy et al., 2016), or unimanual ball rotation (Nojima et al., 2012). In two studies (Reissig et al., 2014; Kumru et al., 2016), a dot was used as a visual target for finger movements. Notably, neither study found increases in ipsilateral excitability that were significantly different when compared to viewing the active hand, however subjects were not instructed to use feedback about movement error to drive performance improvements and behavior was not measured so it is unknown whether subjects responses were indeed heavily reliant on the visual target. The departure from the typical reliance on visually-defined targets for assessment of sensorimotor control is surprising and provides strong scientific premise to empirically study MVF in a motor control framework.

Visual targets are a staple of motor control/learning paradigms (Sarlegna and Mutha, 2015), and have a robust modulatory effect on sensorimotor networks (Koski et al., 2002; Turella et al., 2012). Evidence from neuroimaging suggests that the brain network involved in MVF is considerably overlapped with two fronto-parietal networks known to be more responsive to target-directed actions: the network for spatial attention (Michielsen et al., 2011b; Mehnert et al., 2013; Wang et al., 2013a,b) and the action observation network (AON; Rosén and Lundborg, 2005; Sütbeyaz et al., 2007; Matthys et al., 2009; Ramachandran and Altschuler, 2009; Hamzei et al., 2012; Nojima et al., 2012; Howatson et al., 2013). It is therefore plausible, that MVF combined with a task that requires movements to be performed toward a visually defined target, may have advantages over a paradigm that does not involve an explicit visual target (e.g., see discussion in Arya and Pandian, 2013; Arya et al., 2015). This has never been explicitly tested, but such a finding could help reconcile the discrepancy noted in the MVF literature and advance our understanding of how to best administer MVF. The clinical implications of these findings are important because it remains unknown whether it is sufficient to simply move under MVF conditions, or if one has to engage in a visual target-directed task, in order to optimally use mirror feedback to drive neurophysiological changes. The primary aim of this investigation is to test the interaction of mirror (vs. veridical) visual feedback and the presence (vs. absence) of visual targets during training on M1 excitability of the hemisphere ipsilateral to the trained hand. A secondary aim is to track the time course of CSE modulation to better understand the response to MVF exposure. To address these aims, we developed a virtual reality (VR) environment in which unilateral hand movements performed toward a visual target or a self-determined position (no visual target) can be virtually projected as movements of the same or opposite hand. We measured MEPs throughout training in the M1 ipsilateral to the trained hand and hypothesized that the combination of MVF with visual target-directed movements will lead to significantly greater facilitation of CSE in the ipsilateral hemisphere.

## Materials and Methods

### Subjects

Ten healthy adults (4 female; mean age 27.1 ± 6.3 years) participated in the study after providing written and verbal informed consent in accordance with the Declaration of Helsinki. All protocols were approved by the Institutional Review Board of Rutgers Biomedical Health Sciences. All subjects were right-handed according to the Edinburgh handedness inventory (Oldfield, 1971), free of neurological or orthopedic conditions that could interfere with the experiment, and met inclusion/exclusion criteria to receive TMS (Keel et al., 2001).

### Setup

**Figure 1:**
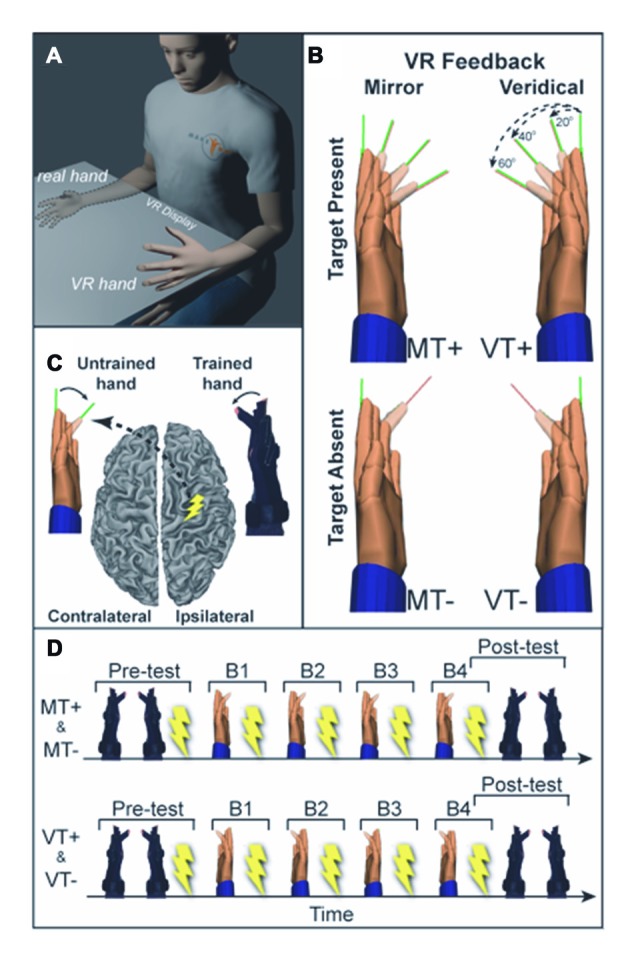
**(A)** Experimental setup. Training (right) hand movements are displayed as movement of a virtual left hand in the mirror visual feedback (MVF) condition. **(B)** Four conditions were used: mirror feedback without targets (MT−), Mirror feedback with targets (MT+), Veridical feedback without targets (VT−), Veridical feedback with targets (VT+). **(C)** Transcranial magnetic stimulation (TMS) was delivered to the ipsilateral (right) motor cortex (M1) and motor evoked potentials (MEPs) were recorded in the untrained (left) hand. **(D)** Prior to and following training participants underwent behavioral testing and TMS assessment of corticospinal excitability (CSE). Training was divided into four blocks (B1–4), each followed by TMS assessment.

Subjects sat with their hands and forearms hidden from view under an LCD and viewed real-time visual feedback of hand motion displayed as VR rendered hand models actuated by kinematic data streaming from data gloves worn on each hand (Figure 1). The VR setup was developed with Virtools (Dassault Systems) and a VRPack plugin that communicates with an open source VR Peripheral Network interfaced with a fiber-optic data glove (5DT-16MRI) measuring 14 finger joint angles (August et al., 2006; Adamovich et al., 2009a,b). Subjects were given 1-min to acclimate to the virtual environment, during which the right and left gloves activated the right and left hands, respectively, without the presentation of targets.

### Task

Subjects were visually cued to flex the right index finger (at the metacarpophalangeal, MCP joint), pause, and return to the start position. Subjects were given 1 s to make the movement and 3 s to return to the start position and await the next trial. Subjects completed four 2-min blocks of 30 movements (B1–B4), separated by 1-min rest periods to allow for TMS assessment.

Visual feedback conditions comprised a 2 × 2 study design with two levels of feedback (Veridical (V), Mirror (M)) and two levels of visual target (Target present (T+), Target absent (T−)). Each subject completed four experimental protocols described by the 2 × 2 design: veridical feedback with visual targets (VT+); veridical feedback without targets (VT−); MVF with visual targets (MT+); and MVF without visual targets (MT−). Under veridical feedback conditions, VT+ and VT−, the actuated virtual hand corresponded to the same side as the moving hand (moving the right hand actuated the right virtual hand). Under MVF conditions, MT+ and MT−, the actuated virtual hand was on the opposite side of the moving hand (moving the right hand actuated the left virtual hand). Conditions including visual targets, MT+ and VT+, required fast and accurate flexion movements to targets presented as thin lines (pseudorandom 3-target repeat (20°, 40°, 60°)). This single joint movement of the index finger isolated the muscle of interest (first dorsal interosseous, FDI), reducing possible confounds from compensatory contraction of muscles not targeted by TMS. Target absent conditions, MT− and VT−, required subjects to make fast non-targeted flexion movements to the middle of the perceived range of motion. The following specific instructions were given to each group.

Target present conditions: “Starting with your hands flat (fingers aligned with your palm), when given the cue to “move”, a green line will appear indicating the target angle. You will make a single smooth fast index finger flexion movement to align the red line extending from the index finger to the green line target. Do not correct your movement within the trial. Make a single movement, and hold the end position until the cue to “return”. The goal is to move as quickly and accurately as possible to the target”.

Target absent conditions: “Starting with your hands flat (fingers aligned with your palm), when given the cue to “move” you are to make a single smooth fast index finger movement to the middle of your perceived range of motion. Make a single movement, and hold the end position until the cue to “return””.

### Electromyographic (EMG) Recording

Surface EMG (Delsys Trigno, 2 kHz) was recorded from the untrained (left) FDI and abductor digiti minimi (ADM) muscles.

### Neuronavigated Transcranial Magnetic Stimulation (TMS)

To ensure accurate coil positioning within and across sessions, frameless neuronavigation (Advanced Neuro Technology) was used to co-register the subjects’ head position to a 3D cortical surface rendering of a canonical high-resolution anatomical MRI scan. The TMS coil (Magstim, 70 mm figure of eight coil) was held tangential to the scalp, with the handle posterior 45° off the sagittal plane inducing a posterior-anterior current in the brain (Littmann et al., 2013). CSE was defined by the size of the motor evoked potential (MEP), quantified as the peak-to-peak amplitude of the EMG signal during a window from 20 ms to 40 ms following the TMS pulse. The location of the cortical hotspot for the untrained (left) FDI was identified by performing a coarse mapping of the right precentral gyrus hand knob area (Yousry et al., 1997; Weiss et al., 2013) to find the loci yielding the largest FDI MEP (Koski et al., 2004; Sollmann et al., 2013). Resting motor threshold (RMT) was determined for the hotspot as the minimum intensity required to elicit MEPs >50 μV in the FDI muscle on 50% of six consecutive trials (Butler et al., 2005). TMS measures were taken at rest (background EMG was monitored to confirm), while subjects directed vision towards a centrally located dot. CSE of the M1 ipsilateral to the trained hand was assessed by collecting 13–15 MEPs (stimulator intensity set to 110% RMT, 4 s ±10% inter-trial-interval) prior to training (Pre) and following each of the four training blocks (B1–4), during 1-min rest periods.

### Behavioral Assessment

To assess if performance was modified by training with MVF, subjects completed a single block of 30 movements to 20°, 40° and 60° targets in pseudorandom order without feedback of the ipsilateral VR hand (VR hands remained stationary). This testing block was performed for the trained (right) and untrained (left) hand prior to the TMS assessment at “Pre” and after the TMS assessment at Block 4 (B4).

### Data Analysis

All data were analyzed off-line with custom written MATLAB software (The MathWorks). Given the single joint nature of the task, only the index finger MCP was used for further analysis. Index finger MCP joint angle data were filtered (4th-order Butterworth: 10-Hz low pass) and marked for movement onset and offset defined as the time at which the angular velocity exceeded and fell below 5% of peak angular velocity for >60 ms. Endpoint error was calculated as the absolute value of the difference between the target angle and the MCP angle at movement offset.

#### Task Kinematics

Reaction time (cue-to-movement onset), movement time (movement onset-to-offset), movement amplitude, peak velocity and endpoint error during training were each analyzed with a rmANOVA with factors Feedback (M, V), Target (T+, T−) and Block (B1–4).

#### Behavioral Assessment

Learning and transfer were quantified as the pre to post reduction of error in the trained and resting hands, respectively. To test for changes in learning and transfer, endpoint error was analyzed with a rmANOVA with factors of Feedback (M, V), Target (T+, T−), Time (Pre, B4) and Hand (Untrained (left), Trained (right)). Significant main effects and interactions were explored using *post hoc* pairwise comparisons with Bonferonni adjustment for multiple comparisons.

#### Corticospinal Excitability

Each subject received 13–15 (mean ± 1 STD, 14.51 ± 0.45) stimulations per block. MEPs were excluded if the average root mean square EMG in the 50 ms preceding TMS exceeded 50 μV (Kumru et al., 2016), if MEP amplitude was found to be greater or less than three standard deviations from the within block mean, or if extraneous subject behavior (such as a sneeze, cough, or movement) was noted during the trial. Across all subjects, blocks, and conditions, a total of 3.03% of stimulations (0.44 ± 0.39 per block) were removed from analysis. An average of 14.07 ± 0.63 MEPs per block were included in the analysis (no single block contained less than 11 MEPs). MEPs were analyzed with a rmANOVA with factors of Feedback (M, V), Target (T+, T−) and Block (Pre, B1–4). Significant main effects and interactions were explored *post hoc* with one-way rmANOVAs for each condition with a single factor of block (Pre, B1–B4). Testing to determine individual block differences from PRE was performed using a Dunnett’s test. When necessary, the Greenhouse–Geisser correction was used to adjust for violations of sphericity. Effects were considered significant at *p* < 0.05. All data are reported as the mean ± SEM.

## Results

### Task Kinematics

**Figure 2:**
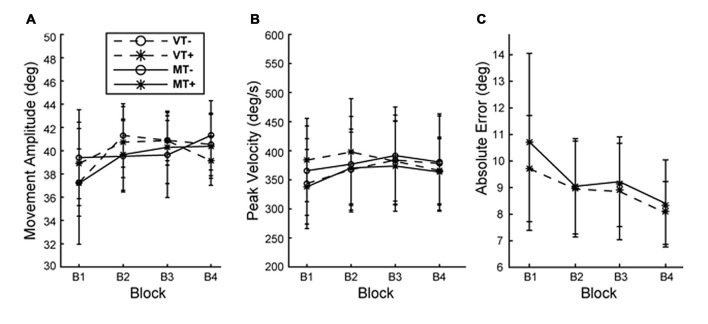
**Behavior during training. (A)** Movement amplitude and **(B)** movement velocity did not differ with condition or block. **(C)** Absolute end point error significantly decreased with block but was not different between MT+ and VT+ conditions. Error bars represent ±1 SEM.

Across all conditions and blocks subjects exhibited a mean reaction time (movement cue to movement onset) of 358.9 ± 42.6 ms and mean movement time (movement onset to movement offset) of 238.3 ± 40.47 ms. A 3-way rmANOVA with factors of Feedback (M, V), Target (T+, T−) and Block (B1–4) on was run on outcomes: reaction time, movement time, movement amplitude and peak velocity. A significant main effect of block was found for reaction time (*F*_(3,27)_ = 10.625, *p* < 0.001) indicating subjects responded faster over time, however there were no main effects of feedback or target and no significant interactions indicating the increase in reaction time was not different between conditions. No significant main effects or interactions were found for movement time, amplitude and peak velocity indicating that movement vigor was comparable across conditions and training blocks. This suggests that behavioral and neurophysiological effects are unlikely to be attributed to differences in performance during training (Figure 2). A 2-way rmANOVA with factors of Feedback (M, V) and Block (B1–4) was used to compare error reduction during training for the visual target directed conditions (MT+, VT+). A main effect of Block (*F*_(3,27)_ = 13.532, *p* = 0.003) indicated that, as would be expected, error was significantly reduced during training for conditions involving a visual target, independent of feedback.

### Behavioral Assessment

**Figure 3:**
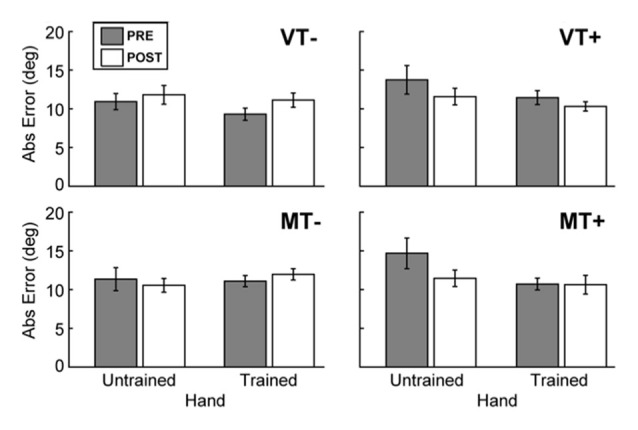
**Behavioral assessment**. The effects of feedback on movement accuracy during PRE/POST behavioral testing. The presence of visual targets (MT+ and VT+) was associated with a significant decrease in error for the trained (right) hand, and the untrained (left) hand. There was no evidence to indicate that feedback affected intermanual transfer. Error bars represent ±1 SEM.

A 4-way rmANOVA, used to examine transfer of learned behavior to the untrained hand, revealed a significant main effect of Hand (*F*_(1,9)_ = 6.004, *p* = 0.037) and a significant interaction of Target × Time (*F*_(1,9)_ = 11.928, *p* = 0.007) for absolute endpoint error. To probe hand specific effects we ran 3-way rmANOVAs (Feedback (M, V), Target (T+, T−), Time (Pre, Post)) for each hand individually. A significant Target × Time interaction was noted for the trained hand (*F*_(1,9)_ = 6.034, *p* = 0.036), and the untrained hand (*F*_(1,9)_ = 13.436, *p* = 0.005). As Figure 3 shows, *post hoc* paired *t-tests* between Pre and Post measures for each hand and condition were not significant, indicating that feedback (mirror or veridical) did not appreciably influence inter-manual transfer.

### Corticospinal excitability (CSE)

**Figure 4:**
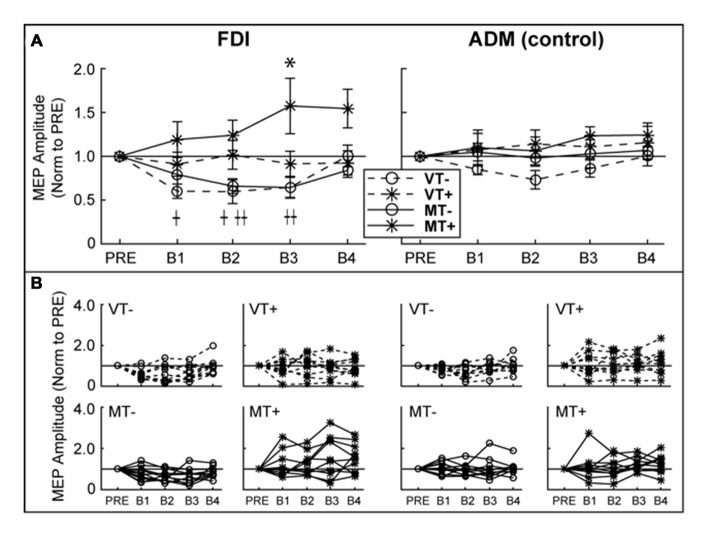
**Ipsilateral M1 CSE**. MEP amplitudes are expressed as a ratio to PRE measurement. **(A)** Group level. There was a significant decrease in first dorsal interosseous (FDI) MEP amplitude relative to baseline in VT− (B1, B2) and MT− (B2, B3). FDI MEP amplitude was significantly increased in MT+ (B3). There were no significant effects in the abductor digiti minimi (ADM). An asterisk, †, or †† indicate a significant within condition difference (*p* < 0.05) relative to PRE for MT+, VT− and VT+, respectively. **(B)** Individual subject data comprising the group data shown in **(A)**. Error bars represent ±1 SEM.

RMT (% Maximum Stimulator Output) did not differ between days (VT−: 55.8 ± 15.0; VT+: 56.0 ± 13.4; MT−: 53.4 ± 12.2; MT+: 53.0 ± 12.8; *F*_(3,27)_ = 1.911, *p* = 0.152), suggesting that the subjects’ baseline neurophysiological state remained consistent across testing sessions. As shown in Figure 4, CSE of the ipsilateral (to the trained hand) M1 gradually increased in the MT+ condition, and decreased in the non-target conditions regardless whether the feedback was veridical or mirror. A 3-way rmANOVA on FDI MEPs revealed a significant main effect of Target (*F*_(1,9)_ = 15.810, *p* = 0.003), and a significant Feedback × Target × Block interaction (*F*_(4,36)_ = 3.230, *p* = 0.023). Individual one-way rmANOVAs for each condition across blocks revealed significant effects for VT− (*F*_(4,36)_ = 4.491, *p* = 0.005), MT− (*F*_(4,36)_ = 4.067, *p* = 0.008) and MT+ (*F*_(4,36)_ = 2.851, *p* = 0.038). Dunnett’s *post hoc* comparisons of each block relative to “Pre” levels confirmed a significant 57.3 ± 31.6% increase for MT+ in B3 (*p* = 0.025), a significant decrease for VT− in B1 (39.9 ± 8.2%, *p* = 0.012) and B2 (40.3 ± 13.6%, *p* = 0.028), and a significant decrease for MT− in B2 (34.1 ± 8.3%, *p* = 0.017) and B3 (35.7 ± 12.1%, *p* = 0.003). No significant changes in CSE were noted for the uninvolved ADM muscle, suggesting that the mirror-induced effects were homotopic to the trained muscle.

## Discussion

We tested the effect that pairing visual target directed training with MVF has on M1 excitability ipsilateral to the trained hand. Participants completed four sessions comprising a 2 × 2 factorial design in which feedback (Mirror, Veridical) and the presence of a visual target (target present, target absent) during a right index-finger flexion task were systematically manipulated in a virtual environment.

### MVF Did Not Improve Transfer to the Untrained Hand

Behavioral testing indicated that the presentation of a visual target during the task significantly decreased error at post compared to the target absent condition. The trained and untrained hands behaved differently, but were not differentially affected by MVF. Though there was a trend towards improvement, MVF did not significantly improve performance in the untrained hand at post-testing (B4), indicating the absence of MVF effects on inter-limb transfer. These results are in agreement with several studies which also did not find an effect of MVF on inter-limb transfer (Carson and Ruddy, 2012; Reissig et al., 2015a,b). Studies that found significant MVF related inter-manual transfer utilized a complex task such as unimanual ball rotation (Nojima et al., 2012; Rjosk et al., 2015), though inter-manual transfer resulting from MVF training did not exceed transfer resulting from viewing the active hand (Reissig et al., 2015a). The task in the present study was likely too simple to distinguish effects of MVF on inter-manual transfer. It is worth pointing out that the lack of inter-manual transfer does not diminish the therapeutic potential of MVF, as it may be a useful technique to induce motor priming in order to increase responsiveness to subsequent training (Thieme et al., 2012; Stoykov and Madhavan, 2015).

### MVF Paired with Visual Target Directed Movements Increased Ipsilateral M1 Excitability

CSE of the hemisphere ipsilateral to the trained hand decreased in non-target conditions and significantly increased (at block 3) only when mirror feedback was combined with a visual target during training. Our results are in agreement with studies that indicate MVF has the potential to facilitate M1 ipsilateral to the moving hand (Garry et al., 2005; Nojima et al., 2012). Likewise, CSE modulation was only found for muscles homologous to those muscles used in the task by the active hand, confirming previous reports that there is homotopic specificity to ipsilateral activation (Garry et al., 2005; Carson and Ruddy, 2012; Kumru et al., 2016). Though we did not assess spinal excitability, previous investigations indicating an increase in ipsilateral CSE have found F-wave amplitude to be unchanged, suggesting a supraspinal mechanism (Nojima et al., 2012). Our data highlight the importance of performing movement to a visual target in order to promote increased ipsilateral M1 excitability using MVF. In target absent conditions (VT−, MT−), a significant decrease of ipsilateral M1 excitability was observed in B1–B2 and B2–B3 respectively. This is congruent with findings that simple unimanual movements such as those utilized in our paradigm are associated with activation of the contralateral hemisphere (Kim et al., 1993; Beltramello et al., 1998) and reduced excitability of the ipsilateral hemisphere, which is thought to prevent involuntary mirror movements (Leocani et al., 2000; Liepert et al., 2001; Duque et al., 2005, 2007; Hübers et al., 2008; Vercauteren et al., 2008).

### Neural Mechanisms of Ipsilateral Activation

A number of neurophysiological mechanisms have been attributed to the activation of the ipsilateral M1 via MVF training. These include interhemispheric interactions associated with cross limb transfer, activation of a bilateral AON, increased spatial attention to the inactive limb, and the resolution of sensorimotor error. We discuss our findings in the context of these neurophysiological mechanisms below.

#### Cross-Activation

Vigorous motor tasks can evoke bilateral M1 activation via interhemispheric disinhibition and/or cross-facilitation between motor and pre-motor areas (Muellbacher et al., 2000; Strafella and Paus, 2000; Stinear et al., 2001; Perez and Cohen, 2008). This manner of interhemispheric M1 modulation, sometimes also labeled as cross-activation, has long been described as a chief mechanism underlying cross-education of strength training (Carroll and Bandura, 1985; Lee and Carroll, 2007; Carroll et al., 2009). A recent investigation combining high-vigor ballistic acceleration training with mirror feedback found that bilateral cortical excitability increased equally when directly viewing the active hand or viewing the mirror reflection of the active hand (Reissig et al., 2014). The authors imply that the movement itself is a more important facilitator of cross-education and ipsilateral excitability, than visual feedback of the movement (Reissig et al., 2014). They temper their argument, however, by explaining that visual feedback may not have been necessary for behavioral improvement and that a task in which visual feedback is required for improvement will more likely show effects of MVF. Despite the plausibility for a role of cross activation in MVF, studies using paired-pulse TMS to probe interhemispheric inhibition changes did not find modulation of interhemispheric inhibition as a potential mediator of MVF (Läppchen et al., 2012; Nojima et al., 2012), except for one study which noted more inhibition with MVF (Avanzino et al., 2014). In the present study, task vigor was minimal and equal across conditions, and movement effort did not increase with training in a way that would be expected to significantly increase excitability of the hemisphere contralateral to the trained hand (though admittedly CSE of M1 contralateral to the trained hand was not directly measured). Therefore, the presence of a feedback dependent effect on ipsilateral excitability despite similar movements across conditions indicates cross-activation is unlikely to be the source of increased ipsilateral excitability in this experiment. An alternative parsimonious explanation is that ipsilateral M1 excitability may be modulated during MVF by extrinsic inputs from cortical areas involved in visuomotor processing.

#### Action Observation Network

Numerous investigations have reported that fronto-parietal regions that make up an AON (Grafton et al., 1996; Rizzolatti et al., 1996; Iacoboni et al., 1999; Buccino et al., 2004a,b; Rizzolatti and Craighero, 2004; Fogassi et al., 2005), those activated during both action execution and observation of movement, may be involved in modulating ipsilateral M1 excitability with MVF (Matthys et al., 2009; Michielsen et al., 2011b; Hamzei et al., 2012; Saleh et al., 2014) (see Caspers et al., 2010 for a review of AON regions). Critical to this hypothesis, and in direct contradiction to speculations of cross-activation, is the recent finding that M1 excitability is lateralized to the viewed hand, rather than to the active hand (Nojima et al., 2012). Together these studies propose that observation of the reflected hand may provide the visual input necessary to excite ipsilateral motor areas (Stefan et al., 2005, 2008; Ramachandran and Altschuler, 2009). The presence of a visual target during action observation seems to trigger selective activation of sensorimotor cortices (Enticott et al., 2010; Donne et al., 2011; Turella et al., 2012; Vesia et al., 2013) and the anterior intraparietal sulcus (Hamilton and Grafton, 2006; Van Overwalle and Baetens, 2009; Ramsey and Hamilton, 2010a,b). The presence of a visual target in the observed action during imitation has also been associated with increased activity in bilateral inferior frontal, premotor and motor cortices (Koski et al., 2002). Compelling results of an fMRI investigation from our group using a target dependent MVF task, indicated significant activation of the intraparietal sulcus in addition to sensorimotor areas (Saleh et al., 2014). When the results of the present study are viewed using an AON-based hypothesis to interpret MVF related cortical changes, it is plausible that the use of a vision dependent target directed task with MVF is critical for the activation of sensorimotor cortices.

#### Error-Based Learning

Intertwined with reported involvement of the AON is the hypothesis that excitability of motor cortices under MVF is induced by sensorimotor conflict between proprioception and visual feedback relative to the static hand (Deconinck et al., 2015). The addition of a visual targets to MVF training introduces a second form of sensorimotor conflict driven by the error between the intended motor action and observed behavior. It is well established that reduction of sensorimotor errors through error-based learning has the potential to increase CSE of the hemisphere contralateral to the trained hand (Bagce et al., 2012, 2013). Though no direct study of error-based learning under MVF conditions has been conducted, previous studies in which sensorimotor conflict during MVF was modified by delaying (Lee et al., 2015), altering (Senna et al., 2015), or providing intermittent feedback (Kang et al., 2012) of the mirror image of the moving hand have indicated an enhanced ipsilateral response with increased discordance. Though no discordance was induced in the present investigation, training resulted in significant systematic reduction of error. While the reduction of sensorimotor error has been most often associated with cerebellar mechanisms (Shadmehr et al., 2010), numerous investigations have described the parietal cortex (including areas known to overlap with the MVF network) to be involved in the association of target location with hand position and therefore integral for the reduction of sensorimotor errors (Mountcastle et al., 1975; Prablanc et al., 2003). This overlap in networks offers the possibility of interactions between MVF resolution and error-based learning.

#### Attention

It cannot be overlooked that areas of the AON and error-based learning network also belong to a network associated with spatial attention. Increased activation of these areas as well as MVF-related activity of the insular, precuneus and cingulate cortices are associated with increased attentional demands that are known to be modulated by goal-oriented behavior (Fink et al., 1999; Adamovich et al., 2009a; Michielsen et al., 2011b; Wasaka and Kakigi, 2012; Wang et al., 2013a). The possible effect of attentional demands on MVF is illustrated in the conflicting findings of two recent articles using EEG assess MVF in which finger flexion and extension movements were either equally intentional (Debnath and Franz, 2016) or differed in intention (one being more automatic; Praamstra et al., 2011). When movement phases differed in intention MVF affects were found to be stronger for the intentional movement, however when movements were equally intentional (requiring similar attention demands) no difference was seen between movement phases. This result emphasizes the possible effects the level of attending to the mirrored hand may have on MVF processing. It is highly likely that in this study the presence of a visual target increased attending to the mirrored hand and may be in part responsible for increased excitability in MT+ condition.

### Timing of Excitability Changes

It remains unknown if increased excitability in the MT+ condition was due to greater attending to the target, activation of AON, or modulation in response to sensorimotor error. Limited interpretation of which systems may be involved can be drawn from the time course of excitability changes. It is logical that target-directed movement and mirror feedback would modify attention in different ways. It might be expected that mirror (vs veridical) feedback might modulate the focus of attention while targeted (vs non-targeted) movements would increase overall attentional demands of the task. Comparing VT− and MT− conditions, attending to the mirrored hand alone did not result in a significant increase (vs Pre) in iM1 excitability during training, however did shift the time course of decreased excitability. Attentional shifts involved in low-level salience of the mirrored hand would be expected to be greatest early in the training and then diminish. It is possible that these low-level salience effects are responsible for the lack of significantly decreased CSE in B1 of the MT− condition in comparison to VT−. It could be concluded that the additional demands of attending to a targeted action alone are enough to reduce movement related suppression of ipsilateral excitability as shown in the comparison of VT− and VT+. However, the addition of these effects would produce an early increase in excitability that diminishes with time, and would not be expected to produce the delayed increase in excitability seen in MT+.

Activation of the AON would be expected to have a neuromodulatory effect relative to the level of agency the subject felt over the virtual hand that could get stronger with time (Adamovich et al., 2009a). Ownership of the hand would be expected to increase with mirror feedback and ownership of the movement would be expected to increase with the addition of the target. Increased ownership of the movement from the addition of visual targets may prevent the decrease in ipsilateral excitability seen in target absent conditions (VT−, MT−). The combination of mirror feedback and targeted movements may instill ownership over movements of the opposite hand, and therefore explain the delayed increase in excitability in MT+.

In target present conditions (VT+, MT+) subjects learned to reduce error during training and demonstrated reduced error at post behavioral testing. Our group has previously shown that CSE increases as a function of learning (Bagce et al., 2013). Therefore, the influence of networks for error-based learning may in part be responsible for the absence of reduced excitability seen in the target free conditions (VT−, MT−). However, learning to reduce target error during training and improvement on behavioral testing did not significantly differ between VT+ and MT+ conditions, and therefore would not be expected to produce the differences observed in iM1 excitability. The delayed increase in ipsilateral excitability seen in the MT+ condition may be linked to the establishment of a control policy (Huang et al., 2011) associated with repetition following discordance resolution of both mirror feedback and target error.

### Limitations

Although the presence of visual targets was found to enhance MVF induced ipsilateral CSE changes, there are several methodological considerations that may limit interpretations of the current study. Subjects may have applied an intrinsic proprioceptive target in the target absent conditions (MT−, VT−). Even if this was the case, the current data still indicate that mirror feedback has a very different effect on M1, as a function of whether movements are performed to an explicit visual target vs. an internally generated non-visual target. The majority of MVF studies use repetitive or continuous tasks. The movement dynamics and motor planning required for a discrete targeted task as was used in current study, and the types of continuous non-targeted tasks used previously differ and may limit comparisons between the current study and previous investigations. Target present (T+) and target absent (T−) conditions differed not only in the presence/absence of a visual target (as intended), but also in the number of targets; subjects were required to move to one of three different visual targets while training in the T+ condition, and only one internal target (if an internal target was used) in the T− condition. This arguably presents a difference in task difficulty between the two conditions. It is therefore plausible that increased M1 excitability resulted not only from increased processing demands created by the use of visual target, but also by the additional task and attentional demands of having to process multiple targets. This is supported by the findings of two investigations in which the interaction of task complexity and MVF was explicitly tested (Verstynen et al., 2005; van den Berg et al., 2011), however neither study manipulated task complexity in terms of number of targets, preventing a direct comparison. Lastly, although we found significant effects, the number of subjects was relatively small, so in the future larger groups of subjects should be studied to validate the timing of iM1 modulation by MVF and visual target directed movements.

## Conclusion

Elucidating the specific neurophysiological mechanism by which MVF promotes activation of the ipsilateral cortex is beyond the scope of this study; however, an agnostic view of the responsible mechanism does not detract from the clinical importance of the findings. Despite potential differences in the cortical substrates, each of these networks has been previously illustrated to be modulated by the presence of a visual target during training. More research is warranted to determine the specific influence that the use of visual targets has on mirror feedback of hand movement before the recommendation can be made that practitioners of MVF therapy utilize visual target directed tasks to maximize changes in ipsilateral CSE.

## Author Contributions

MY, ET and SVA: conception and design of research; MY and TM: performed experiments; MY, ET, SVA and TM: analyzed data; interpreted data; edited and revised manuscript; approved final version of manuscript; MY and ET: prepared figures; drafted manuscript.

## Funding

This work was funded in part by National Institute of Neurological Disorders and Stroke (NIH) grants F31NS092268 (MY), R01NS085122 (ET), National Institute of Child Health and Human Development (NIH) R01HD58301 (SVA) and National Institute on Disability, Independent Living, and Rehabilitation Research NIDILRR grant 90RE5021 (SVA).

## Conflict of Interest Statement

The authors declare that the research was conducted in the absence of any commercial or financial relationships that could be construed as a potential conflict of interest.
